# Emerging Roles of Focal Adhesion Kinase in Cancer

**DOI:** 10.1155/2015/690690

**Published:** 2015-03-31

**Authors:** Yu-Ling Tai, Lih-Chyang Chen, Tang-Long Shen

**Affiliations:** ^1^Department of Plant Pathology and Microbiology, National Taiwan University, Taipei 10617, Taiwan; ^2^Department of Medicine, Mackay Medical College, New Taipei City 25245, Taiwan; ^3^Center for Biotechnology, National Taiwan University, Taipei 10617, Taiwan

## Abstract

Focal adhesion kinase (FAK) is a cytoplasmic nonreceptor tyrosine kinase that enables activation by growth factor receptors or integrins in various types of human cancers. The kinase-dependent and kinase-independent scaffolding functions of FAK modulate the authentic signaling and fundamental functions not only in cancer cells but also in tumor microenvironment to facilitate cancer progression and metastasis. The overexpression and activation of FAK are usually investigated in primary or metastatic cancers and correlated with the poor clinical outcome, highlighting FAK as a potential prognostic marker and anticancer target. Small molecule inhibitors targeting FAK kinase activity or FAK-scaffolding functions impair cancer development in preclinical or clinical trials. In this review, we give an overview for FAK signaling in cancer cells as well as tumor microenvironment that provides new strategies for the invention of cancer development and malignancy.

## 1. Introduction

Cancer signaling emanated from the interaction between cancer cells and tumor microenvironment is critical for cancer development. Integrins are essential bidirectional transmitters in regulating the physical link and signal communication between the inside and the outside of the plasma membrane. Upon integrins engagement with extracellular matrices (ECMs), integrins cluster together on the plasma membrane to ensure the efficient recruitment and activation of various molecules such as adaptor proteins (e.g., p130Cas and Crk), nonreceptor tyrosine kinase (i.e., Src family kinase and focal adhesion kinase), small GTPases (e.g., Rho, Rac, and Cdc42), and cytoskeletal proteins (e.g., talin, vinculin, and paxillin) by forming intracellular specialized complexes and structures named as focal adhesions (or focal contacts) [1]. Utilizing varied signaling proteins within focal adhesions, integrin-mediated signaling enables transmitting cell adhesion signaling as well as tuning the reorganization of cytoskeleton, important for tumor progression, such as tumor angiogenesis and metastasis.

In response to cell adhesion, activation of focal adhesion kinase (FAK) is prominent followed by initially recruited to focal contacts and subsequently autophosphorylated on its Tyr397 to participate in integrin-mediated signaling and functions [2–4]. The FAK nonreceptor tyrosine kinase bears a central kinase domain flanked by an N-terminal FERM (band 4.1 and ezrin/radixin/moesin homology domain) domain and a C-terminal region containing a FAT (focal adhesion targeting) domain and several proline-rich motifs [5], which allows transducing extracellular signals through tyrosine phosphorylation onto a diverse of intracellular molecules in the interior of a cell in both adhesion-dependent and growth factor dependent manners. Specifically, in line of integrin activation, the FAT domain of FAK enables targets FAK onto focal adhesion sites via interactions with other focal adhesion complex proteins, such as paxillin, vinculin, and talin. Consistent with this scenario, we have deciphered an inhibitory mechanism of FAK activation in which the intramolecular interaction between the FERM and kinase domains confers FAK toward an inactive conformation, and the release of this autoinhibition rendered by upstream integrin signaling (i.e., cell adhesion) and/or growth factor signaling in a proximal fashion allows the kinase domain of FAK accessible to numerous catalytic substrates essential for its activation and downstream signaling events [6–8]. Subsequently, the autophosphorylation of FAK on Tyr397 creates a high-affinity binding site for Src homology 2 (SH2) domain-containing proteins, such as Src family kinases, phosphoinositide 3-kinase, phospholipase C, and growth factor receptor-bound protein 7 (Grb7) [9–12], thereby relying the upstream signal on versatile downstream signaling pathways. Moreover, the binding of Src family kinases onto the phospho-Tyr397 of FAK contributes to the promotion of FAK kinase activity and signaling as a result of additional tyrosine phosphorylations on several tyrosine sites, including Tyr407, Tyr576, Tyr577, and Tyr925 of FAK [5]. In fact, the phosphorylation of FAK on Tyr576 and Tyr577 by Src leads to a steric effect on preventing an intramolecular interaction between the aminoterminal FERM domain and the kinase domain within FAK [13]. On the other hand, phospho-Tyr925 of FAK provides a docking site for growth factor receptor-bound protein 2 (Grb2), leading to activation of a RAS-MEK/ERK cascade [14, 15]. In addition, the scaffolding functionality of FAK through its phospho-tyrosine sites and two proline-rich motifs (mainly located within C-terminus) has been observed and elaborated in attribution with targeting a certain array of signaling proteins to focal adhesion sties in response to specific integrin activation [16]. Given the sophisticated regulated mechanism of FAK activation and signal transmission, a myriad of cellular and pathophysiological functions enable modulated in a coopted manner stemming from integrin and/or growth factor activation. Indeed, via recruiting and phosphorylating numerous signaling proteins, FAK empowers cell migration and modulates cell proliferation, adhesion, apoptosis, and differentiation in response to cell adhesion and mitogen stimulation [5, 17], implicating in controlling a wide range of processes of tumor [17]. Inevitably, the mechanistic nature of FAK activation and signaling has been intensively studied to highlight it as a potential target for anticancer therapeutics.

**Figure 1:**
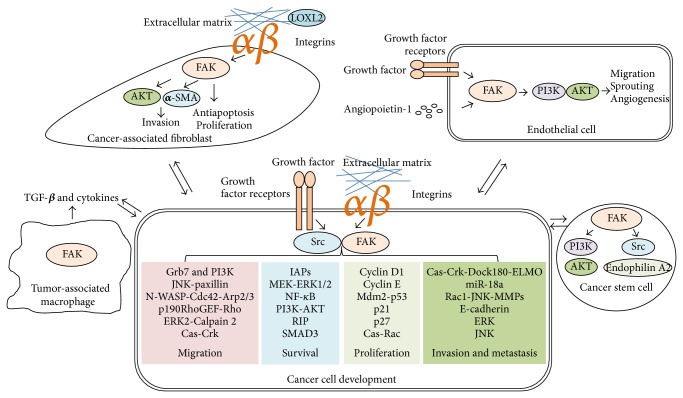
Model for FAK signal transduction in cancer cells and tumor microenvironment. The activation of FAK principally initiated by integrin engaged with ECMs and also by growth factor receptors enables regulating cell survival, proliferation, migration, invasion, and metastasis in relation to cancer development. Subsequently, the autophosphorylated (on Tyr397) FAK/Src complex empowers tyrosine phosphorylation cascades in modulating versatile signal pathways. For example, FAK modulates endophilin A2 phosphorylation by Src or PI3K-AKT signaling in cancer stem cells. In endothelial cells, vascular endothelial growth factor-A (VEGF-A)/VEGF or angiopoietin-1 signalling regulates FAK-mediated PI3K/AKT activation to promote migration, sprouting, and angiogenesis. FAK also regulates the expression of growth factors or cytokines in tumor-associated macrophages to facilitate cancer progression. In response to LOXL2 stimulation, FAK affects the *α*-SMA expression and AKT signaling to control invasion, antiapoptosis, and proliferation in cancer-associated fibroblasts.

Tumor microenvironment, a mixture of varied cell types as well as secreted cytokines and deposited ECMs, is indispensable for tumor progression and metastasis [18, 19]. Upregulation of integrins and FAK is often observed to correlate with the progression of tumor development, implying the integrin/FAK signaling involved in regulation of tumor development [20]. Moreover, the activation of FAK enables modulation by growth factor stimulation. In this review, we provide an overview of FAK signaling in cancer cell biology and discuss how FAK signal transduction controls the cancer development and progression as summarized in Figure 1. In addition, we also summarize the potent anticancer drugs in relation to FAK-mediated signaling.

## 2. FAK in Cancer Cells

### 2.1. FAK Signaling in Cancer Cell Survival and Proliferation

#### 2.1.1. FAK Regulates Cancer Survival

FAK-mediated signaling plays a critical role in the regulation of cancer cell survival. In opposite to cell adhesion, anoikis is a form of programmed cell death due to the disruption of cell-ECM interactions, in which FAK activity is lost and, therefore, results in cell apoptosis [21, 22]. In contrast, often overexpression of FAK in cancer cells seems to attribute the resistance of cell detachment-induced cell death (i.e., anoiksis). Indeed, in these cancer cells, the increased FAK/Src complex confers the activation of both PI3K-AKT and MEK-extracellular signal-regulated kinase 1/2 (ERK1/2) signal transductions, thereby enhancing the ability of cancer cell survival and growth in a cell detached condition [23]. In addition, several other upstream signals also contribute to FAK-mediated anoikis resistance in cancer cells. For example, transforming growth factor-*β* (TGF-*β*) induces the activation of FAK and AKT via SMAD3 and p38 MAPK, respectively, which, in turn, results in anoikis resistance and tumor promotion [24]. In squamous cell carcinoma (SCC), fibronectin-mediated integrin *α*V activation leading to FAK phosphorylation at Tyr397 prevents suspension-induced or tumor suppressor p53-mediated anoikis [25]. As expected, disruption of the organized fibronectin structure enables anoikis of SCC, presumably losing FAK phosphorylation and ERK activation. Moreover, overexpression of FAK has been shown to block the caspase-3-mediated apoptosis; conversely, inhibition of FAK leads to apoptosis in cancer cells [26]. Hence, knockdown of FAK expression by RNA interference promotes anoikis and further inhibits pancreatic cancer metastasis* in vivo* [27].

Antiapoptotic property is also essential for FAK in prevention of cancer programmed cell death. Actually, overexpression of FAK apparently links to the activation of PI3K-AKT signal transduction and promotes the expression of NF-*κ*B-mediated inhibitor-of-apoptosis proteins (IAPs), which warrants apoptotic inhibition by blocking caspase-3 cascade in human leukemia [28]. Likewise, the phosphorylation of NF-*κ*B and activation of PI3K/AKT signaling modulated by FAK disable the tumor necrosis factor-*α*- (TNF-*α*-) induced apoptosis [29]. Moreover, FAK also reportedly binds to the death domain kinase receptor-interacting protein (RIP), a component of death receptor complex in the programmed cell death, by which FAK is capable of suppressing apoptosis by blocking the function of death domain of RIP [30]. These studies indicate that an essential role for FAK activation and FAK-mediated signal transduction is to direct the fate of cancer cells by preventing the programmed cell death.

#### 2.1.2. FAK Regulates Cancer Proliferation

Cell proliferation is a process of the increment of cell number as a result of cell division and growth. Discovery of the expression and tyrosine phosphorylation of FAK highly correlated with cell cycle progression by modulating cell cycle-relative molecules [31] highlights FAK functioning as a key regulator in promoting cancer proliferation. Eventually, overexpression of FAK enables increasing cyclin D1 expression and decreasing cyclin-dependent kinase (CDK) inhibitor p21 expression, thereby accelerating G1 to S-phase transition. Conversely, the competition of FAK localization and function out from focal contacts by a dominant-negative FAK mutant inhibits cell cycle progression at G1 phase [32]. Furthermore, FAK has been elaborated to modulate the E26 transformation-specific (ETS) binding site resided within the cyclin D1 promoter, which, in turn, regulates the transcriptional activation of cyclin D1 and leads to promoting cell cycle progression [33]. Indeed, overexpression of FAK also exits cell cycle from G1 phase in glioblastoma. Consistently, the promotion of cell cycle progression by FAK is mediated by enhanced expression of cyclin D1 and cyclin E in concert with reduced expression of CDK inhibitors p27^Kip1^ and p21^Waf1^ [34].

The tumor suppressor p53 triggers lots of antiproliferative processes, such as DNA repair, apoptosis, and cell cycle arrest [35]. On the other hand, FAK promotes cell cycle progression by the blockage of tumor suppressor p53-mediated apoptosis and the inhibition of p53 transcriptional activity [36]. Mechanically, it has been proposed that FAK might exert its FERM domain as a scaffold to stabilize p53 and ubiquitin E3-ligases Mdm2 in the nucleus, which, in turn, enhances Mdm2-dependent p53 ubiquitination and subsequently leads to p53 polyubiquitination and degradation. In other words, the nuclear FAK's FERM domain enables mediating p53 turnover in regulation of cell proliferation and survival [37]. Accordingly, mammary tumor malignancy and progression originated from the loss of p53 expression or overexpression of a dominant-negative p53^R270H^ mutant are impeded upon FAK deletion [38]. Thus, FAK modulates cancer cell proliferation by either regulating cell cycle-relative molecules or promoting tumor suppressor turnover.

### 2.2. FAK Signaling in Cancer Cell Migration, Invasion, and Metastasis

#### 2.2.1. FAK Signaling-Mediated Cytoskeleton Remodelling in Cell Migration

Cytoskeletal remodelling is critical for cancer cell migration, therefore indispensable for cancer metastasis. FAK signaling which resulted from ECM-induced integrin clustering intimately involves in the reorganization of cytoskeleton and cell motility. In response to specific chemoattractant signals, such as ECMs and growth factors, protrusions are formed at the leading edge by polymerization of actin filaments towards the cell plasma membrane, following the formation of focal adhesion complexes where activated integrins render the recruitment and activation of FAK [39, 40]. Through the FAK/Src complex formation and kinase activity, cell migration is profoundly affected by the subsequent phosphorylated p130Cas in promoting the formation of Cas/Crk complex [41], myosin light-chain kinase- (MLCK-) mediated focal adhesion disassembly [42], c-Jun N-terminal kinase- (JNK-) mediated phosphorylation of paxillin in facilitating cytoskeleton reorganization [43], interactions with PI3-kinase and/or Grb7 in governing intracellular signaling related to cell motility [44, 45], and so on. Beside the essential role for the focal adhesions assembly, integrin-mediated FAK signaling governs focal adhesion turnover (adhesion dynamic) to ensure a dynamic and complex process of the membrane protrusion during cell migration. For example, enhanced extracellular signal-regulated kinase 2 (ERK2) activity mediated by the formation of the FAK/Src complex at focal adhesion sites leads to protease Calpain 2 activation, which subsequently allows Calpain 2 to cleave the molecules, for example, FAK and talin, at the focal adhesion sites in facilitating focal adhesion turnover in motile cells [46–48].

FAK also impacts on the remodelling of actin cytoskeleton and stabilization of focal adhesions through its effect on small GTPases [17]. Small GTPases (i.e., RhoA, Rac1, and Cdc42) function as critical roles in cytoskeleton reorganization [49]. RhoA affects cell-cell or cell-ECMs interaction by inducing the change of cytoskeleton, Rac1 plays a role in membrane ruffling via driving actin polymerization, and Cdc42 involves in the formation of filopodia through initiating actin filament assembly [49–51]. Given to bearing GAP activity for RhoA and Cdc42 [52], Graf, a FAK interacting protein, empowers FAK signaling in regulation of actin cytoskeleton reorganization. Alternatively, the formation of the FAK/N-WASP complex leads to the activation of Cdc42, followed by engagement of Arp2/3 and actin filament formation [53, 54]. Moreover, FAK binds to p190RhoGEF which leads to the activation of Rho and maturation of focal adhesions in association with tumor migration [54]. These studies provide evidence for FAK and its signaling in substantially controlling Rho GTPases and subsequently vital for cytoskeletal reorganization.

#### 2.2.2. FAK Promotes Cancer Invasion

Cancer invasion toward the surroundings at the primary site is a critical event for tumor malignancy. Numerous numbers of evidence indicate that FAK predominately involves in the promotion of tumor invasion, implicating that FAK is a potential target for anticancer therapeutics. In a coopted manner of receptor tyrosine kinase and integrins, the formation of the FAK/Src complex reinforces FAK signaling through p130Cas to recruit the Crk-Dock180-ELMO complex in promoting the activation of Rac1, which, in turn, activates JNK protein and results in MMPs production for ECM proteolysis [17, 55]. The consequence of MMP-mediated ECM proteolysis is pivotal for cancer invasion that can be facilitated by FAK-mediated signaling in response to mobile signals. Nevertheless, prior to cell invasion, cancer cells* per se* need to proceed with a developmental and morphological alternation called epithelial-mesenchymal transition (EMT), a process to transdifferentiate epithelial cells into motile mesenchymal cells [56]. Epithelial cadherin (E-cadherin) is the major molecule resided within epithelial adherent junctions. During the occurrence of EMT, degradation of E-cadherin is accordant with the disruption of adherent junctions, which allows the release of cell-cell restriction and, therefore, promotes cancer invasion [49]. Reportedly, the phosphorylation of FAK at Tyr407 and Tyr861 by the FAK/Src complex drives the interruption of E-cadherin-mediated epithelial adherent junctions [57–59]. Consistently, transforming growth factor-*β*- (TGF-*β*-) induced EMT because of downregulation of E-cadherin is mediated by the activation of FAK in a Src dependent manner [60]. In the process of cancer invasion, the activation of FAK in cancer cells could transmit numerous downstream signal pathways in regulating a variety of cellular events, including cytoskeletal remodelling and EMT, to control cell fate.

#### 2.2.3. FAK Promotes Cancer Metastasis

In agreement with its essential role in cell migration, FAK is inferred to be crucially involved in cancer metastasis due to the fact that overexpression and activation of FAK are often clinically associated with cancer metastasis [54, 61, 62]. Actually, the Tyr397 phosphorylation and kinase activity of FAK are substantiated to be important for the invasive phenotype as well as cancer metastasis [63]. Intrinsically, FAK kinase activity has been emphasized in the promotion of cancer metastasis by regulating the expression of MMP9 and urokinase plasminogen activator [64]. In addition, increased FAK expression can rescue the miR-7 negative effect on tumor migration and invasion [65]. Accordingly, suppression of FAK expression and signaling by RNA interference (RNAi) or dominant-negative mutants of FAK (i.e., FAK-related nonkinase, FRNK) generally enables inhibiting FAK-triggered cancer metastasis [66–69]. The fact is that FRNK expression reducing the FAK phosphorylation on Tyr397 and decreasing the formation of focal adhesions is attributed to its inhibitory effect on cancer metastasis* in vivo*, rather than the FRNK-induced dormancy or apoptosis of cancer cells [70]. Moreover, FRNK expression also disrupts the formation of v-Src/FAK complex, which, in turn, inhibits p130Cas activity and reduces the v-Src-stimulated ERK and JNK kinase activation, leading to impair v-Src-mediated cell invasion and metastasis [71]. On the other hand, using RNAi targeting FAK to reduce the expression of FAK has been observed in reduction of cancer metastasis in cervical lymph node metastasis of tongue cancer [66]. Additionally, although a synergistic effect of FAK and epidermal growth factor receptor (EGFR) is highlighted for their cooperation in the promotion of non-small cell lung cancer metastasis, knockdown of FAK and EGFR expression using short hairpin RNA (shRNA) significantly inhibits cancer metastasis [72]. These observations emphasize the important role for FAK in regulating tumor malignancy by directing cancer invasion and metastasis.

### 2.3. FAK in Cancer Stem Cells

In spite of being controversial, cancer stem cells remain an attractive subject for understanding underlying tumorigenesis and malignancy as well as cancer therapeutics. As defined, cancer stem cells bear the ability to self-renewal, differentiate into versatile cancer cell types, and promote tumor progression [73]. Additional property in maintaining a relatively quiescent and long life span renders cancer stem cells more resistant to current chemotherapies [74]. Recently, deletion of FAK in mammary epithelial cells (MaECs) has been reported to affect breast cancer development, that is, suppression of mammary tumor formation and progression, in the mammary tumor mouse MMTV-PyMV model [75]. In that study, impairment of the cancer stem cell maintenance in FAK ablated MaECs has been taken into account for the decreased tumorigenicity and metastasis of mammary tumors [75]. Indeed, loss of FAK leads to less activation of the PI3K-AKT signaling and, therefore, impedes MaCSC self-renewal and tumorigenicity [76] although a compensatory activity by Pyk2, a nonreceptor tyrosine kinase of the FAK family, might appear [76]. Besides, the role of integrin/FAK signaling in the regulation of cancer stem cells is observed in squamous cell carcinomas [77]. Yet, these studies remain unidentifying the mechanistic nature of FAK and its signaling involved in regulation of cancer stem/progenitor cell population. In this regard, the differential role of kinase-dependent and kinase-independent functions of FAK in mammary stem cells (MaSCs) and luminal progenitors had been explored. The kinase activity is required for the sphere-forming of MaECs and the proliferation of luminal progenitor; in contrast, the kinase-independent function of FAK necessitates in ductal invasion and basal MaSC activity [78]. Consequently, the inhibitor of FAK kinase activity is not efficient in targeting MaSC-like human breast cancer cells compared with luminal progenitor-like basal breast cancer cells [78]. Furthermore, a functional defect of FAK on its Pro-878/881 motif leads to impairing FAK scaffolding in mediating endophilin A2 phosphorylation by Src, thereby leading to profound decreases in epithelial-mesenchymal transition (EMT) and MaCSC activities [79]. Collectively, these findings provide an intrinsic role for FAK in the regulation of cancer stem cells, which entails FAK as a novel target for anticancer therapeutics by intervention of the cancer stem cell activity.

## 3. FAK in Tumor Microenvironment

During cancer progression and malignancy, tumor cells continuously encounter with their surroundings in a dynamic and complicated manner. Nevertheless, tumor microenvironment commonly indicates the solid-state extracellular matrices (ECMs), resided soluble factors, nonneoplastic cells, including cancer-associated fibroblasts, endothelial cells, bone marrow progenitor cells, and tumor-associated macrophages, and so on [18]. The communication between cancer cells and tumor microenvironment is mainly attributed by secreted soluble factors (e.g., growth factors, cytokines, and chemokines) and extracellular matrices remodeling enzymes (e.g., matrix metalloproteinases, MMPs and tissue inhibitor of metalloproteinases, and TIMPs) governing cell-cell and cell-EMC interactions as well as cell signaling events [80].

Tumor microenvironment is an essential and vital niche in support for the developmental process of tumors. As mentioned above, in light of tumor microenvironmental cues, activation of FAK is capable of driving the integrin- and growth factor receptor-mediated signaling inside of a cell to ignite tyrosine phosphorylation cascades and reorganize cytoskeleton of tumor cells. On the other hand, how the FAK of tumor-associated cells resided within the tumor microenvironment activated and contributing to tumor progression is exploring in recent years. Herein, the emerging roles of FAK in the varied cell types of tumor microenvironments will be discussed below.

### 3.1. Integrins/FAK Signaling in Response to ECMs in Tumor Microenvironment

Cumulative deposition and remodeling of ECMs fabricating a stiffer tumor microenvironment usually result in resided tumor cells losing the regular cytoskeletal organization [81]. As cell adhesion receptors, integrins possess specific and varied binding affinities with various ECM molecule compositions to provide a tensional homeostasis in response to the tense states of tumor microenvironments. Authentically, the mechanotransduction caused by the ECM stiffness enables reflecting on integrin-mediated FAK signaling influences tumor progression [82]. Increased lysyl oxidase (LOX) activity has been highlighted responsible for the consequence of ECM stiffness due to its ability for collagen cross-link, which is in accordance with the promotion of cancer malignancy as well as invasiveness and metastasis in varied cancers, including breast cancer cells [83], brain tumor [84], and colorectal cancer progression [85]. In response to the increment of tumor matrix-stiffness, activation of FAK is also correlated with the clustering of *β*1 integrin encountering with the stiffen matrices and then elicits PI3K/AKT signaling [86]. In addition, ECM stiffness initiated by LOX activation is also capable of activating MYC-induced upregulation of miR-18a in need of *β*1 integrin clustering. Thus, reduction of FAK activity by a FAK inhibitor is proved to modulate the expression of miR-18a followed by lowing PTEN expression and elevating PI3K/AKT signaling in promoting tumor invasion and metastasis [87]. Moreover, the activation of FAK through ECM stiffness can modulate the intracellular structure and promote cell cycle progression rather than cell migration [88], in which the FAK/p130Cas/Rac signaling pathway is responsible for S-phase entry in the aid of increased cyclin D1 [88]. ECM stiffness-mediated FAK activation and FAK-regulated tumor biological functions in distinct malignant cancers suggest that the ECM-mediated FAK signal transduction might function as a general and crucial point in tumor developmental processes.

### 3.2. Endothelial Cell FAK Contributes to Tumor Angiogenesis

In addition to tumor cells, FAK also plays an essential role in endothelial cells involved in tumor progression. Tumor angiogenesis as a process for the formation of new blood vessels is an essential step for the development of tumor malignancy. Indeed, FAK signaling is important to regulate angiogenesis in embryonic development as well as pathological angiogenesis (e.g., tumor angiogenesis) [89–92]. The expression and activation of FAK are frequently correlated with endothelial cell survival, proliferation, and migration [93, 94]. For example, VEGF-A/VEGF-receptor 2 signaling induces the FAK/PI3K complex formation by which endothelial cells migration is promoted to facilitate angiogenesis [95]. In addition, FAK phosphorylation mediated by angiopoietin-1 stimulation also enhances endothelial cell sprouting in a PI3K dependent manner [96]. Moreover, FAK-deleted endothelial cells reduce tumor angiogenesis, which is caused by reduction of VEGF-mediated AKT phosphorylation [89]. In tumor-associated endothelial cells, FAK activity is essential for VEGF-mediated tumor extravasation and metastasis [97]. Collectively, FAK contributes to tumor development in diverse mechanisms. Realization of FAK-mediated signaling highlights and provides the opportunities in the development of anticancer therapeutics.

### 3.3. FAK in Tumor-Associated Macrophages

Tumour-associated macrophages (TAMs) play a protumoral role via stimulation of angiogenesis and enhancement of tumor cell invasion, migration, and metastasis. TAMs are also immunosuppressive, preventing tumor cell attack by T cells and NK cells during tumor progression and after recovery from chemotherapy or radiotherapy [98]. FAK signaling is required for macrophage motility induced by integrin engagement [99]. Importantly, inhibition of FAK signaling reduces the infiltration of macrophages into tumor tissues [100–102]. FAK in TAMs is thought to be bioactive; however, how FAK in TAMs regulates TAMs-mediated tumor malignancy is largely unknown. Nevertheless, the followings indicate a potential protumoral role of FAK in TAMs.

TAMs promote tumor growth and distant metastasis by secretion of various kinds of proteins in the paracrine manners that induce tumor epithelial-mesenchymal transition, invasion, migration, and angiogenesis [98]. It is worth to note that the effect of FAK in TAMs on the expression of these proteins. Epithelial-mesenchymal transition is induced in the invading tumor cells by TGF-*β* that is expressed by TAMs and is regulated by FAK in fibroblast cells [98, 103]. The process of tumor migration requires matrix remodeling facilitated by matrix metalloproteinase 9 that is regulated by FAK in follicular thyroid carcinoma cells and fibroblasts [104–106]. In addition, neovascularization is important to tumor growth and metastasis. TAMs stimulate neovascularization by the production of VEGF and IL-8 [98, 107]. The expression of VEGF is regulated by FAK in epithelial cells [108, 109] as well as the expression of IL-8 in fibroblast-like synoviocytes and epithelial cells [110, 111]. In addition to establishment of a favorable microenvironment, TAMs also inhibit the antitumor immune responses through the suppression of cytotoxic functions and the induction of apoptosis in T cells and NK cells. Therefore TAMs attenuate tumor surveillance in the tumor progression and antitumor immunity elicited by tumor-derived antigens released from therapy-mediated dying tumor cells [112]. TGF-*β* expressed by TAMs is regulated by FAK in fibroblast cells and can inhibit T cells effector functions through the induction of regulatory T cells [98, 103]. Taken together, FAK is important to the induction of TAMs through the recruitment of macrophages into tumor tissues and potentially execute protumoral functions through the ability to regulate the downstream gene expression. The potential role of FAK in TAMs is largely unknown and worth to be investigated.

### 3.4. FAK in Cancer-Associated Fibroblast

Cancer-associated fibroblasts (CAFs) within tumor stroma promote cancer development by secreting chemokines or growth factors to govern several oncogenic signals in cancer cells, endothelial cells, and inflammatory cells [113, 114]. FAK had been reported to involve in the regulation of CAFs. Tumor-derived lysyl oxidase-like 2 (LOXL2) promotes the expression of *α*-smooth muscle actin (*α*-SMA) and activates fibroblasts through integrin-mediated FAK activation and AKT signaling [115]. Moreover, inhibition of FAK leads to impairing the phosphorylation of AKT as well as the reduction of the expression of *α*-SMA [115]. FAK activation is also important to prevent the anoikis by providing the antiapoptotic signal in both fibroblasts and epithelial cells, which implies that FAK-mediated aberrant survival signals in normal cells might play some functions in affecting the cancer development [116]. On the other hand, FAK^−/−^ fibroblasts block cell migration; the reexpression of FAK and the kinase activity of FAK in FAK^−/−^ fibroblasts are required for cell migration and invasive phenotype defects [63]. Indeed, loss of FAK in the epidermis had been reported to reduce the malignant progression of skin cancers by enhancing the apoptosis [117]. In addition, the activation of FAK might alter the organization of tumor microenvironment and facilitate the cancer development in pancreatic ductal adenocarcinoma (PDA) [100]. In PDA mouse model, inhibition of FAK impairs cancer cells proliferation and leads to the decreased CAFs recruitment [100]. Based on the study about the prognostic gene-expression signature of CAFs in non-small cell lung cancer, the modulation of focal adhesion signaling might be regulated by integrin and FAK signal transductions [118]. These studies suggest that FAK is vital in the cancer development by regulating the cellular and biological functions of CAFs.

## 4. FAK in the Development of Targeted Therapeutics

Overexpression of FAK has been clinically observed in primary human sarcomas [119], human prostate carcinomas [120], human ovary carcinomas [121], human colorectal carcinomas [122], and breast cancers [123], implicating the role of FAK in cancer development. Experimental evidence also indicates that FAK-mediated signaling and functions intrinsically involve in the development of tumor malignancy, suggesting that FAK is a promising target for anticancer therapies. Eventually, several approaches have been developed to modulate the expression or activation of FAK, including small chemical inhibitors, PF-573,228 and VS-4718, in capable of blocking the autophosphorylation of FAK on Tyr397. According to the aforementioned roles for FAK in cancer, some inhibitors have been launched in phase I clinical trial for anticancer therapies. Besides, targeting specific protein-protein interaction which prevents the transduction of FAK-mediated signaling is an alternative strategy for anticancer therapy. The following will further summarize and discuss recent development and progression of FAK-related anticancer therapies.

### 4.1. FAK in Human Cancers

Several mechanisms have been reasoned for the overexpression of FAK protein in human cancer in correlation with the* fak* gene amplification. Using* in situ* hybridization, increased dosage of the* fak* gene on chromosome 8q24.3 is invariantly observed in the cell lines derived from human cancers of lung, breast, and colon [124]. Moreover, gains in copy number of* fak* gene in invasive squamous cell carcinomas also revealed in correlation with elevation of FAK protein and tumorigenesis [124]. In addition, both primary colorectal cancers and colorectal liver metastases express high levels of FAK mRNA copy number as well as FAK protein expression level [122]. However, elevated FAK expression is not always correlated with gains on* fak* gene amplification. For example, in human head and neck squamous cell carcinoma, not all cases with an amplification of the* fak* gene displayed FAK protein overexpression [125], implicating a sophisticated posttranscriptional regulation involved in FAK expression and functions.

By analyzing the 5′ promoter region of the* fak* gene, NF-*κ*B and p53 transcription factors can bind within the FAK promoter region [126]. However, NF-*κ*B and p53 transcription factors exhibit opposite activities in the regulation of FAK transcription. NF-*κ*B induces FAK transcription, whereas p53 suppresses FAK transcriptional activity [126]. In human breast and colon cancers, FAK expression is significantly increased in tumors containing p53 mutations in comparison to tumors bearing wild-type p53 [127, 128]. Moreover, missense p53 mutations in its DNA-binding domain, that is, V173M, R249M, and R282M, also confer to promote the FAK promoter activity [127]. Furthermore, miR-151 derived from the intron 22 of the* fak* gene which is often coexpressed with its host gene* fak* may also be regulated by p53 transcription factor in human hepatocellular carcinoma [129]. The synergistic effect of miR-151 and FAK can stimulate Rac1, Cdc42, and Rho GTPases signaling, which, in turn, leads to enhancing cancer motility and invasion [129]. These studies imply that FAK expression and its biological functions may be modulated by the transcriptional regulators and its coamplification miR-151.

The upregulation of FAK has been specifically associated with liver metastases of colon cancer, which infers a role for FAK in tumor invasiveness* in vivo* [130]. Several studies suggest that a coordinate modulation of FAK-mediated signals and functions is crucial for tumor malignancy [131–133]. Moreover, the overexpression of FAK and phosphorylation of FAK on Tyr397 is frequently associated with tumor metastasis as well as poor patient prognosis [134–136], indicating a critical role for FAK in tumor progression and malignancy. These studies suggest that FAK could be functioned as a potential prognostic marker and anticancer candidate.

### 4.2. Targeting Kinase Activity of FAK

In agreement with the importance of FAK kinase activity in FAK-mediated signaling and development of tumor malignancy, ATP-competitive kinase inhibitors are capable of binding the ATP-binding pocket of FAK to efficiently block FAK catalytic activity. Their potency, signaling effect, and functional efficacy on tumor malignancies will be discussed as follows.

*PF-573,228*. This compound is a bisamino pyrimidine derivative with the half maximal inhibitory concentration (IC_50_) for the FAK catalytic activity at 4 nM whereas for FAK autophosphorylation on Tyr397 at 30–100 nM. Importantly, PF-573,228 blocks cell migration in concomitant reduction in the disassembly of focal adhesions. In contrast, this compound fails to inhibit cell growth or induce cell apoptosis [137].

*TAE226*. This compound is a bisanilino pyrimidine derivative. TAE226 inhibits the phosphorylation of FAK and the FAK-mediated signaling, such as AKT, ERK, and S6 ribosomal protein in glioma [138]. Subsequently, the inhibition of FAK signaling by TAE226 induces the cell cycle arrest and increases cancer apoptosis. TAE226 also impairs glioma tumor adhesion, migration, and invasion [138]. Besides glioblastomas, TAE226 is capable of accomplishing the detachment and apoptosis of breast cancers but no effects on MCF-10A normal breast epithelial cells [139]. In animal models, TAE226 prolongs the survival rate with breast cancer bone metastasis [140]. TAE226 also suppresses the growth and angiogenesis of oral squamous cell carcinoma in a xenograft mouse model [141]. To be noted, in addition to FAK kinase activity, this compound also blocks the activation of poly (ADP-ribose) polymerase and caspase-3. [139]. Currently, TAE226 has been undergone its efficacy test in the preclinical phase.

*PND-1186 (Also Termed VS-4718)*. This compound is a substituted pyridine reversible inhibitor for FAK activity with an IC_50_ at 1.5 nM* in vitro* [142]. Not only reduction in cell migration but also PND-1186 triggers cancer apoptosis in a 3D environment via blockage of the FAK/p130Cas tyrosine phosphorylation cascade and induction of caspase-3 activation [142]. Consistently, PND-1186 impacts on oncogenic KRAS- and BRAF-stimulated MDA-MB-231 breast cancer growth and metastasis by blocking FAK and p130Cas phosphorylation [143]. In the preclinical phase, PND-1186 displays anticancer efficacy in orthotropic breast cancer mouse models. At present, PND-1186 is under phase I clinical trial.

*PF-562,271 (Also Termed VS-6062)*. This compound is a bisamino pyrimidine derivative as a FAK activity inhibitor with an IC_50_ at 1.5 nmol/L [144]. In therapies for pancreatic ductal adenocarcinoma model, PF-562,271 inhibits migration of cancer cells, cancer-associated fibroblasts (CAFs), and tumor-associated macrophages (TAMs) [100], indicating that PF-562,271 may exert a novel mechanism in inhibiting cancer metastasis by altering the tumor microenvironment. PF-562,271 has been on the progress of the clinical trial targeting advanced solid tumors [145].

### 4.3. Alternative Mechanism to Block FAK-Mediated Cellular Functions

Protein-protein interactions are a fundamental event for signal transductions and involved in the development of tumor malignancy. Eventually, recruitment and interaction with the intracellular domain of growth factor receptors (e.g., insulin-like growth factor receptor-1, IGF-1R and vascular endothelial growth factor 3, VEGFR-3) are required for FAK activation essential for regulations of diverse signal transductions in relation to cancer development. These observations prompt a possibility to interrupt the interaction between FAK and growth factor receptors for potential anticancer therapeutics. For example, a small molecular compound chloropyramine hydrochloride (called C4) targets the interaction between FAK and VEGFR-3, which, in turn, reduces the phosphorylation of both proteins, leading to decreasing the proliferation and causing the apoptosis in breast cancer [146]. Besides, a compound INT2–31 targets the FAK/IGF-1R complex and further effectively inhibits AKT phosphorylation, leading to decreasing melanoma cells proliferation and tumor growth* in vivo* [147]. Furthermore, rather than disruption with growth factor receptors, the 5′-*O*-tritylthymidine (called M13) compound blocks the formation of the FAK/Mdm-2 complex; subsequently, it activates p53 and caspase-8, leading to increase detachment and apoptosis in breast and colon cancers [148]. Likewise, a small molecule compound R2 can disrupt the interaction between FAK and p53, which, in turn, increases p53 transcriptional activity, contributing to inhibit tumor growth [149]. These compounds target FAK scaffold which is a critical strategy to block the FAK-mediated cancer development.

Alternatively, RNA interference (RNAi) is an emerging means for anticancer therapies. Using RNAi to inhibit FAK expression, it can lead to the death of anoikis-resistant tumor cells and also reduce the metastasis of pancreatic cancer in a mouse model [27]. Simultaneous inhibition of both FAK and EGFR expression by specific shRNAs exhibits a synergistic effect on lung cancer metastasis* in vivo* [72]. In addition, ectopic expressions of miR-138 and miR-135 targeting the FAK 3′UTR reportedly suppress FAK-mediated tumor growth and invasion as well as drug sensitivity [150]. miR-7 also serving as a direct regulator of FAK expression by targeting the FAK 3′UTR, as expected, enables suppressing the FAK-mediated malignancies in aggressive breast cancers, such as proliferation, anchorage independent growth, migration, and invasion [65]. Besides, the dominant-negative mutants of FAK (i.e., FAK-related nonkinase, FRNK) may also provide an alternative way to block FAK-triggered cancer malignancy [66–69]. These strategies via targeting FAK mRNA or FAK-mediated protein-protein interaction/signal transduction advance the development of FAK-targeted therapeutics.

## 5. Conclusions

The kinase-dependent function and kinase-independent scaffolding ability of FAK are equivocally essential for cancer development. Moreover, the multifunctional characteristics of FAK have been highlighted to modulate numerous signal transductions in governing the activities and functions of the tumor microenvironment, cancer cells, and/or cancer stem cells (Figure 1). However, the complicated cross talk and regulatory loops modulated by FAK between the tumor microenvironment and cancer cells/cancer stem cells remain to be deciphered. In lines of clinical observations, overexpression of FAK at both transcriptional and translational levels in human varied cancers implies that FAK could be a prognostic marker and a potential anticancer candidate for target therapies. In fact, several small compounds targeting the catalytic activity, protein-protein interactions, and even RNAi of FAK have been launched for conducting clinical trials and some clarifications remain to be verified such as drug sensitivity for specific types or stages of cancers, appropriate dosages, side effects, and the toxicity [151]. Beyond this, the resistance of cancers to FAK inhibitor should be pondered deeply as the next problem. Several studies indicate that the combination of FAK inhibitor with chemotherapy or other anticancer molecules as a combinatory therapy for cancers effectively attenuates cancer development [146, 152]. Nevertheless, the synergistic effect of the combination of FAK inhibitor and chemotherapy or other anticancer molecules remains to be investigated for its possible regulatory mechanisms in the anticancer therapy. Collectively, the profound progress during the past decades in the molecular and cellular mechanisms and cancer biology of FAK has provided much basic and clinical medical knowledge for targeting FAK as a potential anticancer therapeutic strategy.
